# Attributions of Blame in Stranger and Acquaintance Rape: A Multilevel Meta-Analysis and Systematic Review

**DOI:** 10.1177/1524838020977146

**Published:** 2020-12-07

**Authors:** Sofia Persson, Katie Dhingra

**Affiliations:** 1 School of Social Sciences, Leeds Beckett University, United Kingdom

**Keywords:** victim blame, acquaintance rape, rape myth acceptance, ambivalent sexism, multilevel meta-analysis

## Abstract

**Background:**

Victim blame, particularly in cases of acquaintance rape, presents an obstacle to criminal justice. Past research indicates that acquaintance rape results in more blame than stranger rape. However, there are inconsistencies in these findings (e.g., whether there is a linear relationship between victim blame and relationship closeness), partly due to methodological variation.

**Objectives:**

To examine the effect of victim–perpetrator relationship on victim blame, how this effect is impacted by rape myth acceptance (RMA) and ambivalent sexism (AS), and to establish what the methodological quality is of studies.

**Synthesis method:**

Studies were synthesized through a multilevel meta-analysis using the Metafor package in R (version 2.4-0), synthesizing findings from 47 individual studies. Studies compared victim blame between stranger and acquaintance rape, in isolation or in conjunction with RMA and AS, and were identified through a database search.

**Results:**

The review found higher levels of blame in acquaintance as compared to stranger rape, with a medium effect size. This effect was not moderated by RMA. AS was not included as a moderator in the meta-analysis, but the review indicated that benevolent sexism may be a particularly relevant variable.

**Implications:**

Future research should examine the relationship between AS and victim blame. The current review contributes to the evidence base on victim blame in rape cases by suggesting that methodological limitations can account for some of the past mixed findings in this area, particularly in a lack of consistency in vignette details. It is recommended that future sexual assault research uses rigorous methodology and increases transparency of research processes.

## Sexual Assault

Globally, one in five women will experience sexual assault at some point in their life (Garcia-Moreno et al., 2005; Rape Crisis England and Wales, 2019). Only a minority of rapes, however, are reported to the police, with international estimates ranging from 15% to 24% reporting rates (Krahé et al., 2008; Morgan & Kena, 2018; Rape Crisis England and Wales, 2019). A significant barrier to reporting is a lack of trust in the criminal justice system, and studies have documented that victims/survivors anticipate that the police will respond to reporting of sexual assault with disbelief (Cohn et al., 2013). Similarly, victims may also feel ashamed and guilty following a sexual assault, particularly if they anticipate negative reactions from others, including friends and family (Cohn et al., 2013; Thompson et al., 2007). Concerns about negative social reactions and perceived stigma from others may be particular salient for women who have been assaulted by an acquaintance (Krahé et al., 2008). These figures are a serious cause for concern, as it has been suggested that the relative impunity with which men can carry out sexualized crime against a large number of women constitutes a miscarriage of justice (Barr & Pegg, 2019; Gravelin et al., 2018), and calls for further investigation into the factors that influence attributions of victim blame in rape cases.

## Victim–Perpetrator Relationship

The vast majority (up to 90%) of rape cases are perpetrated by someone the woman knows, who will, in most cases, be a current or former intimate partner (Black & McCloskey, 2013; Office for National Statistics, 2018; Rape, Abuse & Incest National Network, 2020). Most research distinguishes between stranger and acquaintance rape, where the former refers to a sexual assault perpetrated by someone whom the woman has not previously encountered. The definition of acquaintance rape is considerably broader; this includes perpetrators ranging from someone the woman has just met, to a partner or a spouse (Grubb & Turner, 2012). Past research suggests that victims who know their perpetrator in any capacity are typically assigned more blame than victims of stranger rape (Gravelin et al., 2018; Hockett et al., 2016). Possible mechanisms behind this difference in blame assignment include the perception that women who are willingly alone with men should expect sexual attention and traditional notions of rape not being possible, or severe, within heterosexual relationship, as aided by the delayed criminalization of marital rape.

However, as noted by Grubb and Turner (2012) and Grubb and Harrower (2009), findings related to the impact of the victim–perpetrator relationship on victim blame have been inconsistent. They further note that studies have yielded particularly inconsistent findings with respect to relationship proximity; sometimes there is a direct correlation between closeness and victim blame (i.e., where someone who is assaulted by their partner is blamed more than someone assaulted by a friend), and sometimes there is not. In their review, Grubb and Harrower (2009) call for further investigation into the potential cause(s) of these inconsistent and contradictory findings (e.g., methodological differences between studies, moderator effects, etc.), particularly within the context of expanding the acquaintance rape category, to allow for further distinctions in known-perpetrator scenarios. Additionally, Grubb and Turner (2012) note that future research should investigate how the victim–perpetrator relationship interacts with variables such as rape myths and gender role attitudes.

Therefore, to account for the above considerations, this article examines rape myth acceptance (RMA) and ambivalent sexism (AS) as two constructs that may have an impact on the effects of the victim–perpetrator relationship on victim blame and explains some of the inconsistencies discussed above. As few studies measured AS, this construct is included in the review, but not in the meta-analysis. RMA is included in both the meta-analysis and the review.

## RMA

First conceptualized by Burt (1980), rape myths are a set of persistent and widespread beliefs and attitudes that serve to exonerate the perpetrator and blame the victim of rape. RMA is the degree to which someone holds these attitudes. Rape myths include beliefs about the victim’s character, appearance, and behavior; the motivations and behavior of the offender; and the situational factors surrounding the offense (e.g., the area, time of day, method; Burt, 1980; Sleath & Bull, 2012) and can be broadly categorized into four categories: blaming the victim; exonerating the perpetrator; the belief that rape is not very common or serious; and the belief that only certain types of women are raped (Gerger et al., 2007; Lonsway & Fitzgerald, 1994). Rape myths have been found to be persistent across community and professional samples, and men consistently exhibit higher levels of RMA than do women (Gerger et al., 2007; Hine & Murphy, 2019; Persson et al., 2018; Suarez & Gadalla, 2010). Crucially, a large evidence base has found that RMA correlates with victim blame in rape cases by positioning women as the cause of rape (for a review, see Grubb & Turner, 2012), and it seems to play a particularly important role in assigning blame in acquaintance rape cases (Gravelin et al., 2018). However, no studies to date have systematically examined whether RMA can explain the inconsistencies in past research into victim blame in stranger and acquaintance rape or how it may relate to AS in this context.

## AS

AS consists of benevolent sexism (BS) and hostile sexism (HS), two different, but complimentary constructs that together form an ambivalently sexist attitude toward women (Glick & Fiske, 1996). Glick and Fiske (1996) posit that BS encapsulates attitudes that may on the surface seem beneficial to women, such as women being pure and moral and in need of protection from men. In reality, these attitudes serve to differentiate women from men and emphasize women’s responsibility to serve as “gatekeepers” of male sexuality. On the contrary, HS is the more overt form of sexism, which encompasses negative attitudes toward women and their capabilities, such as women being deceitful and untrustworthy (Glick & Fiske, 1996; Lee et al., 2010). As argued by Chapleau et al. (2007), the above forms a “stick and carrot” system, which keeps women within the patriarchal order, as it justifies male domination while rewarding women who comply with its rules. HS and BS correlate strongly with each other (Glick & Fiske, 2001), but may have different relationships with variables such as victim blame ( Viki & Abrams, 2002). As noted by Chapleau et al. (2007), RMA generally correlates with HS but not necessarily with BS. It is likely that this is because both RMA and HS encapsulate a zero-sum game of heterosexual relationships (i.e., the perception that the advancement of a gender out-group’s rights [i.e., women’s rights] would be at the expense of a gender in-group’s rights [i.e., men’s rights]), and negative views about women.

At the core of AS is the categorization of women into “good” and “bad” women. Studies have shown that the women viewed as bad are seen as being deserving of adversarial consequences such as rape by those who are high in sexism, but it often results in a self-perpetuating argument, as women who have been raped can be categorized as bad simply by having been raped (Harrison et al., 2008; Viki & Abrams, 2002). This aspect of AS also relates more directly to RMA, as a central tenant of RMA is that only certain women (i.e., those putting themselves at risk through drinking or sexual promiscuity) are raped, and women who violate traditional gender roles are assigned more blame for a rape by those high in RMA (Grubb & Turner, 2012). It is, therefore, possible that AS can explain some of the past inconsistencies relating to the victim–perpetrator relationship and victim blame. The current review, therefore, includes studies that measure participants’ AS using the Ambivalent Sexism Inventory (ASI; Glick & Fiske, 1996).

## The Current Study

Two recent reviews are notable in having done much to systematically expand knowledge about attributions in rape cases; Hockett et al. (2016) examined the impact of rape myth consistency and gender on attributions of blame in rape cases, and Gravelin et al. (2018) outlined the individual, situational, and sociocultural factors that have an impact on victim blame in acquaintance rape situations. While these two papers are informative, the current review and meta-analysis aim to expand on issues not directly covered in these papers, consequently filling a gap in knowledge about attributions of blame in rape cases. The current analysis includes a larger number of studies, as it compares blame attributions in stranger *and* acquaintance rape, rather than between men and women (Hockett et al., 2016), or in acquaintance rape scenarios only (Gravelin et al., 2018). The main contributions of this article to the extant literature are outlined in the following sections.

First, this article examines the moderating impact of RMA as a relatively stable attitudinal variable of the individual, as opposed to viewing it as a situational variable varying according to the rape vignette, which was the approach taken by Hockett et al. (2016). The current conceptualization of RMA is, therefore, in line with how it was originally viewed by Burt (1980) and also by subsequent researchers (e.g., Bohner et al., 2009). Treating RMA as a feature of the individual vignettes rather than an attitudinal variable is problematic, as it assumes that people interpret rape scenarios in identical ways, that is, that one situation is considered high in RMA regardless of who is reading about it. As argued by Bohner et al. (2009), rape scenarios are notoriously ambiguous, meaning that people rely on heuristics to make inferences about who is to blame. These mental shortcuts do not generally rely on facts of the case, but rather on preexisting social attitudes, which include gender roles and rape myths (Bohner et al., 2009). Therefore, measuring RMA as an attitudinal variable rather than stable and objective features of the scenario is truer to the nature of how blame attributions in rape cases are processed.

Second, the review section of this article considers the role of AS. While a small but significant number of studies have examined the role of AS within the context of the victim–perpetrator relationship, there has not yet been a systematic synthesis of this literature. This is surprising, given its interaction with the victim–perpetrator relationship (Abrams et al., 2003). Further, despite the theoretical links between AS and RMA (Chapleau et al., 2007), there has yet to be a systematic examination of the two.

Third, although previous reviews (e.g., Grubb & Harrower, 2009) have provided useful insights into the impact of the victim–perpetrator relationship on victim blame, this article includes a meta-analysis and also assesses the methodological quality of the included studies. It, therefore, seeks to make concrete recommendations for future research in this area, particularly in the context of methodological quality and rigor. Relatedly, this article adheres to Open Science principles, an increasingly relevant paradigm within science. This was achieved through preregistration of the research aims and the use of open access software R (version 3.6.2) for the analysis, with meta-analysis code and the associated data set being available online. All of this material is hosted on the study’s Open Science Framework (OSF) page (Persson & Dhingra, 2020). The current meta-analysis attempts to account for dependency among effect sizes (ESs; resulting from drawing more than one effect from individual studies) by analyzing the data using a three-level meta-analytical approach (Viechtbauer, 2010), which has not been previously undertaken in this context.

## Problem-Intervention-Comparison-Outcome (PICO) Statement

The PICO framework serves as a guide for implementing evidence-based reviews. The objectives of the current review are to examine (a) how victim blame may vary between stranger and acquaintance rape scenarios; (b) how RMA and AS impact this; and (c) what the methodological quality is of the included studies. Participants in the included studies are adult women and men.

## Method

### Protocol

The Cochrane Collaboration’s (Higgins & Green, 2011) guidelines for conducting systematic reviews and meta-analyses were followed during the review process. The manuscript is reported in line with Preferred Reporting Items for Systematic Reviews and Meta-Analyses (2009) guidelines. Research aims and methodology are preregistered on the OSF (Persson & Dhingra, 2020).

### Inclusion and Exclusion Criteria

Included studies were required to compare blame attributions between stranger and acquaintance rape scenarios and to be sufficiently similar to permit a direct comparison. A stranger rape perpetrator was defined as someone whom the victim had not met prior to the assault, and an acquaintance rape perpetrator as someone whom the victim had met prior to the assault. This ranged from a very brief acquaintance to a partner or a spouse. Studies were required to include a measure of victim blame, victim responsibility, or victim guilt. Studies portraying the rape scenario through a vignette or by other means (e.g., fictitious court documents, newspaper story) were included. Correlational studies were included if it was possible to obtain data to facilitate ES calculation. Studies had to be available in English. Where data on participants’ RMA and AS were available, this was also included.

### Search Strategies

The search phrase used to access literature was (“rape” OR “sexual assault”) AND (“blame” OR “responsibility” OR “guilt”) AND (“rape myth acceptance” OR “RMA” OR “ambivalent sexism” OR “hostile sexism” OR “benevolent sexism”). Additional searches without the suffixes were also conducted to reflect the inclusion of papers only comparing type of rape, without RMA, or AS. Keywords were developed based on previous literature (e.g., Gravelin et al., 2018; Grubb & Turner, 2012). The following databases were searched: Web of Science, PsycINFO, MEDLINE, PubMed, OVID, CINAHL, and PsychArticles. ProQuest Theses and British Library EThOS (E-theses online service) were searched to identify potential gray literature, and all authors who were contacted about additional data (see information below for further details) were also asked about any unpublished material they might have. The literature list of other prominent reviews (e.g., Gravelin et al., 2018; Grubb & Turner, 2012; Hockett et al., 2016) were cross-checked to ensure no papers were missed. Search hits were imported into the web application Rayyan (Ouzzani et al., 2016), where they were screened for eligibility.

**Figure 1.**
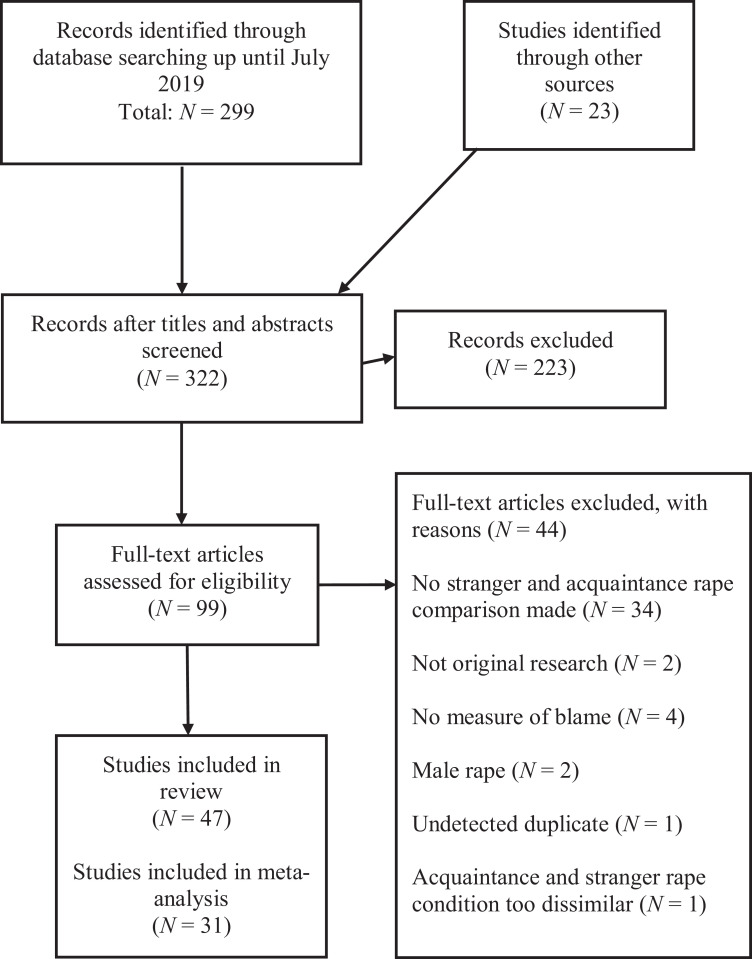
Study flow chart.

The search identified 299 unique records from the database, and an additional 23 studies were added from other sources. From these, 223 records were excluded based on irrelevancy after screening the title and abstract. Ninety-nine records were examined in detail for eligibility, where 52 were eventually excluded. Exclusion reasons included the following: the paper not comparing stranger and acquaintance rape; original research findings not presented; target outcome variable not examined; a focus on male rape; the paper being an undetected duplicate; and the acquaintance and stranger rape condition being too dissimilar (i.e., one contained alcohol consumption and provocative clothing worn by the victim, whereas the other did not). A total of 47 individual studies (derived from 44 publications) were included in the systematic review. Of these, 31 were included in the meta-analysis (with 49 ESs, as some studies reported more than one ES). Where information relating to ES calculations was missing, authors were contacted requesting additional data. Authors were contacted in 16 cases and responded with the requested data in eight of those. In one case, the author was deceased. Finally, one study (Idisis et al., 2007) was excluded from the meta-analysis due to concerns over bias. A flowchart with the study selection process can be found in Figure 1.

### Data Extraction and Coding

A data extraction sheet was developed by the first author. The data extracted were as follows: (a) study and ES identification (study ID and ES ID); (b) demographical study information (name, year, author, publication status, and country of origin); (c) demographical participant information (percentage of females and mean age); (d) vignette details (relationship status in acquaintance rape scenario and type of scale used to assess victim blame); (e) moderator information (RMA score and type of RMA scale); and (f) ES measures (stranger *N*, acquaintance *N*, ES, Standard Error [*SE*], and variance). In addition, a study quality score was added to the data extraction sheet, as further detailed below.

Study quality was assessed using a tool from the Agency for Healthcare Research and Quality (Williams et al., 2010) and modified to include relevant aspects of the current research topic (e.g., level of vignette details, quality of relevant moderators). It included 12 items in total, which covered issues relating to sample selection, group matching, dependent measure and moderator reliability, vignette detail, blinding, approaches to missing data, consideration of confounds, statistical reporting, and analytic methods. Full details on this tool (including scoring guidelines) can be found on our OSF page (Persson & Dhingra, 2020). Studies received a “yes” where criteria were fully met and a “no” where they were not met. Where criteria were partially met, a study received “partial.” Each criterion was also given a score (yes = 2, partial = 1, no = 0) to allow for its inclusion in moderator analyses. The maximum possible quality score (indicating a higher quality paper) was 22. For reliability purposes, 100% of the data were coded independently by the first and second author. Agreement was high (93%), and any disagreements were resolved through discussions between the authors. Too few studies reported data on AS to include this as a variable in the meta-analysis.

### Statistical Analysis

Cohen’s *d* was calculated as the measure of ES of the difference in blame attribution between the stranger and acquaintance rape condition. A positive ES indicated that more blame was attributed to the victim of acquaintance rape, as compared to the victim of stranger rape. Where Cohen’s *d* was not reported in the original study, ESs were either converted from other effects (e.g., *F*) or calculated using the Campbell Collaboration’s Practical Meta-Analysis Effect Size Calculator (Wilson, 2019). As recommended by Assink and Wibbelink (2016), the variance was calculated as *SE*^2^.

Twelve studies provided more than one ES, as participants read more than one acquaintance rape vignette (e.g., used a within-subjects design), therefore violating the requirement of a lack of dependency among ESs in meta-analysis (Cheung, 2014; Rosenthal, 1986). Dependency of ESs normally means that ESs within studies are correlated; this creates an overlap of information and inflates information produced by the analysis, which can result in an overconfidence in its results (Assink & Wibbelink, 2016; Van den Noortgate et al., 2013). While conducting subgroup analysis or aggregating ESs are common responses to this particular issue, both of these reduce the number of effects analyzed in a set, therefore limiting power of the analysis. Assink and Wibbelink (2016) note that this is a particular concern when conducting moderator analyses. An alternative option, which we used in this research, is fitting a three-level meta-analytical structure, which accounts for this interdependency, while allowing flexibility in examining moderators. The analysis considers three levels (variance components) in the model. This includes how ESs vary according to participants (level 1), outcomes (level 2), and studies (level 3; Cheung, 2014). This type of approach produces a robust analysis but has yet to be implemented in a sexual assault context.

The analysis was conducted using the rma.va function in the Metafor package (Viechtbauer, 2010) for the statistical software environment R (R Core Team, 2019). A mixed-effects model was fitted, and estimation was based on the restricted maximum likelihood estimator. The analysis examined the variance distribution over the three levels, the overall effect (i.e., victim blame in stranger as compared to acquaintance rape), and the effects of a number of moderating variables. R code was adapted from Assink and Wibbelink (2016) and Harrer et al. (2019).

Publication bias was examined using a power-enhanced (sunset) funnel plot (Kossmeier et al., 2020), which plots ESs (Cohen’s *d*) against their *SE*s. It also illustrates the power of each study to detect the desired ES. If small study bias is present, the funnel is asymmetrical, as the small studies without a large ES are missing, indicating that only the small studies with large ESs are present.

Categorical moderator analyses were conducted using dummy coded variables, with one category for each variable used as the reference category (Assink & Wibbelink, 2016; Harrer et al., 2019). The analysis compares the reference category to each of the other categories and highlights where effects occur, with an estimation of mean effects. For example, the analysis for victim–perpetrator relationship used the dummy coded variable “casual acquaintance” as the reference category and compared this to the effects of each of the remaining three categories (further details on these below). Continuous moderator analyses estimated mean regression coefficients (βs) for each variable. As studies employed a variety of scales to measure RMA, a standardized average of each mean score was created by dividing the mean score with the maximum score in the relevant scale.

## Findings

### Results of Meta-Analysis

#### Data preparation

Based on recommendations by Snyder et al. (2015), ESs three *SD*s above or below the mean ES were considered outliers and thus excluded. Therefore, one ES comparing rape by a stranger to that of a casual acquaintance (Bendixen et al., 2014) was removed; the final sample of ESs was, therefore, reduced from 49 to 48 ESs. In line with the procedures of Assink and Wibbelink (2016) and past meta-analytical research in this area (Hockett et al., 2016), categorical moderators (level of familiarity and outcome variable) were dummy coded (0 = *absent*; 1 = *present*) to allow for an estimation of mean effects of each category, measured against the other categories. Dummy coded variables included acquaintance rape level of familiarity (acquaintance–just met, casual acquaintance, date/partner/husband, and ex-partner/ex-husband) and outcome variable (victim blame, victim responsibility, and victim guilt). For example, level of acquaintance created four dummy coded variables: acquaintance–just met (0 = *absent*; 1 = *present*), casual acquaintance (0 = *absent*; 1 = *present*), date/partner/husband (0 = *absent*; 1 = *present*), and ex-partner/ex-husband (0 = *absent*; 1 = *present*). RMA, percentage of women in the sample, and year of publication were all treated as continuous moderators.

#### Descriptive features of the studies

All included studies were experimental in nature. Most commonly, participants were presented with a vignette (varying level of acquaintance between victim and perpetrator) describing the rape of a woman by a man and then asked a number of questions relating to victim blame. If AS and RMA were measured, these scales were generally presented to participants prior to the vignettes.

Studies were conducted between 1976 and 2019. A total of 13,872 participants were included in the analysis, and studies had median sample size of 275 (min = 57, max = 863). ESs came from the United States (*N* = 23), Germany (*N* = 10), UK (*N* = 4), Sweden (*N* = 4), Australia (*N* = 3), Norway (*N* = 1), Turkey (*N* = 1), Slovenia (*N* = 1), and Japan (*N* = 1). More than half (54.20%) of the total sample were female, with a mean age of 26.37 (min = 19.21, max = 42.91). Students were by far the most common participant group (*N* = 32), followed by legal professionals (*N* = 8), community samples (*N* = 7), and medical professionals (*N* = 1). The most common relationship in the acquaintance condition was casual acquaintance (*N* = 26), followed by a current partner (*N* = 11), an ex-partner (*N* = 7), and brief acquaintance (*N* = 4). Dependent variables could be categorized as victim blame (*N* = 24), victim responsibility (*N* = 23), and victim guilt (*N* = 1).

#### Main analyses

**Figure 2.**
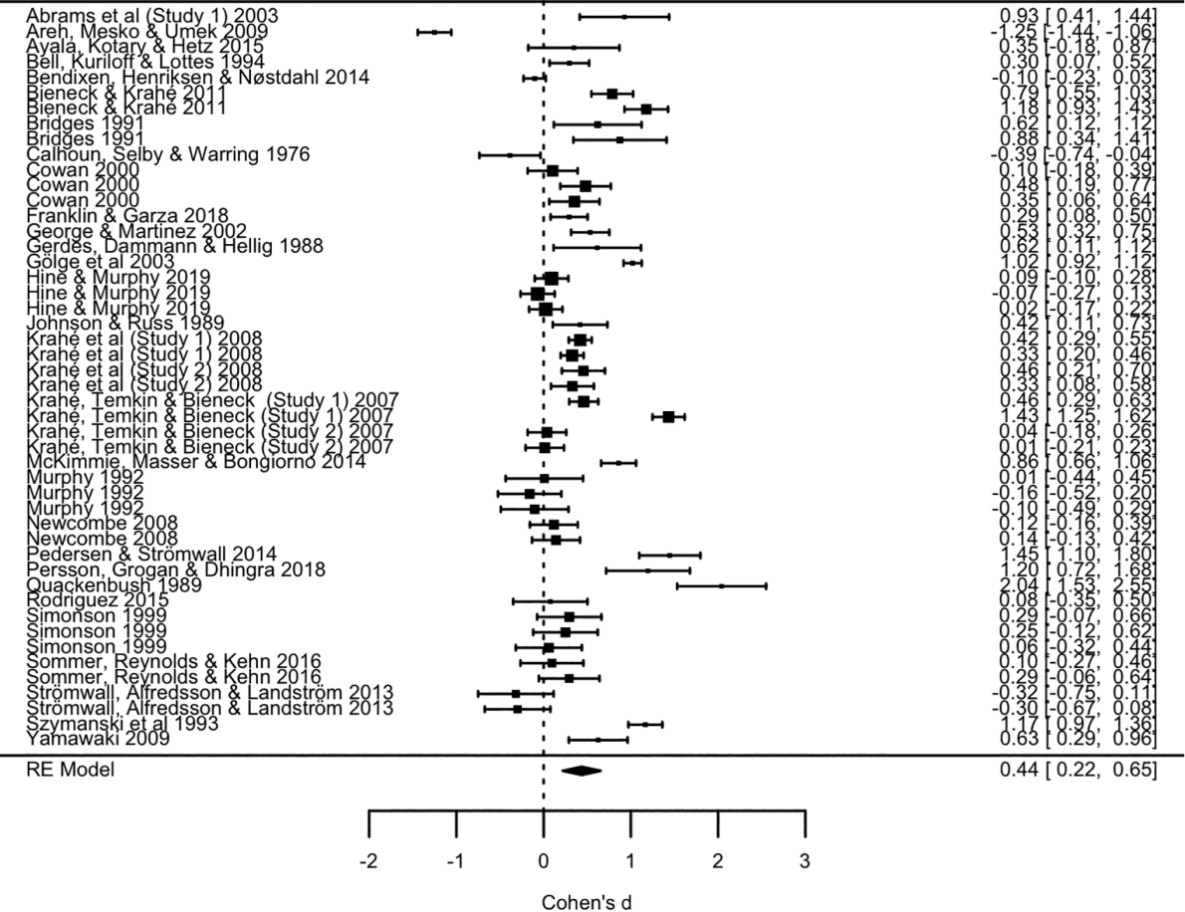
Forrest plot. *Note.* Author names and study dates are listed in the far-left column. Study effect size (ES) and confidence intervals are listed in the far-right column and visually depicted in the middle column. The overall ES (random effects model) is listed and illustrated in the final row.

The first step of the analysis estimated the overall ES of the difference in perceived victim blame between stranger and acquaintance rape (48 ESs from 31 studies). Across all studies, the overall mean effect for the difference between stranger and acquaintance rape in perceived victim blame was medium-sized, *d* = 0.44 (*p* < .001), *SE* = 0.11, 95% CI [0.22, 0.65]. Therefore, there was a significant difference in perceived victim blame, where victims of acquaintance rape were consistently attributed more blame than victims of stranger rape. A forest plot can be found in Figure 2.

The second step of the analysis estimated the difference between within- (level 2) and between (level 3)-study variance components. This was assessed through two separate log-likelihood ratio tests, where the original model was compared to one where the variance at each of the level was fixed. The analyses indicated that there was significant variability (*p*s < .001) between ESs (level 2) and also between studies (level 3), indicating that moderator analyses should be conducted (Assink & Wibbelnk, 2016).

#### Moderator analyses

None of the moderator analyses were significant (see Supplementary Material); the effect of victim-perpetrator relationship on victim blame was not affected by type of relationship, participant characteristics, level of RMA, type of outcome variable (victim blame or victim responsibility), or year of study publication. This is in line with details in the systematic review below.

#### Risk of bias

**Figure 3.**
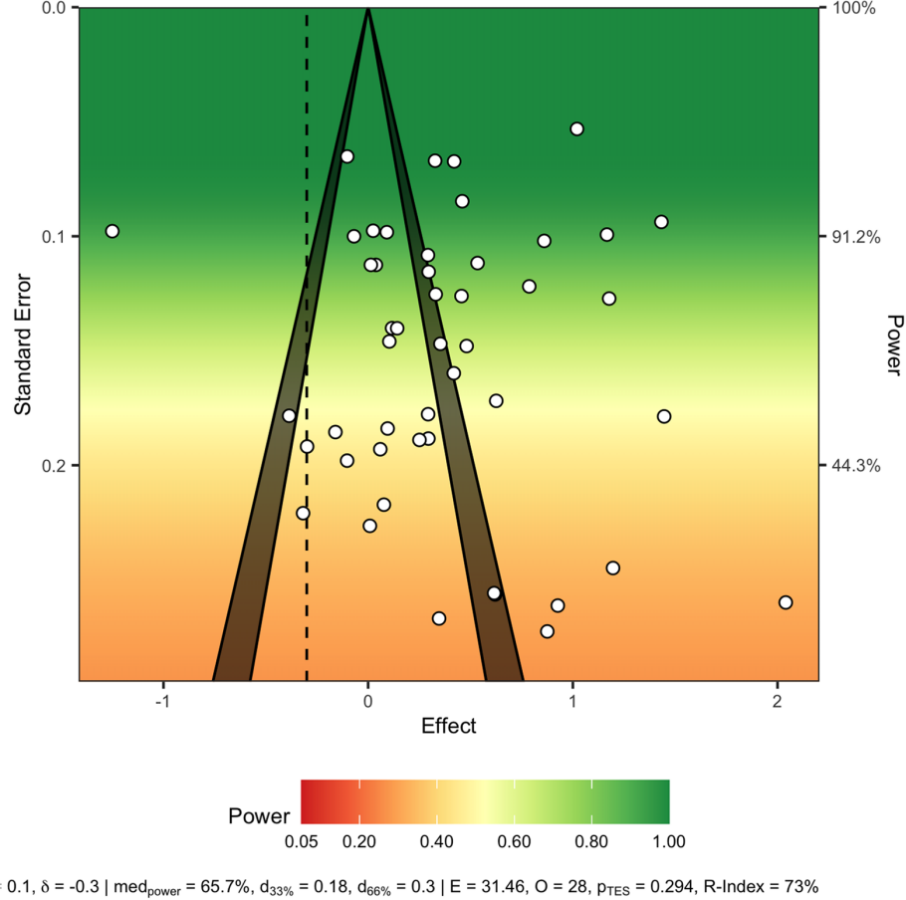
Power-enhanced funnel plot (sunset plot). *Note.* Effect sizes (ESs; *x*-axis) are plotted with corresponding *SE*s (*y*-axis). Study ESs are depicted as circles. If no publication bias exists, all study ESs fall within the middle funnel. Where publication bias exists, study ESs fall outside the funnel, indicating that there are studies with less extreme ESs missing. The plot also displays the power of each study to detect the effect of interest.

Figure 3 depicts the funnel plot for the random effects model.

The sunset funnel plot demonstrates evidence of publication bias, as the funnel is asymmetrical. Although there is considerable symmetry among the majority of the studies, a small number fall well outside the funnel. However, when testing this with an Egger’s regression (Egger et al., 1997), where the *SE*s were entered as a moderator in the main analysis, this did not demonstrate significant publication bias, β = 2.22, *p* = .19, 95% CI [−1.14, 5.57]. Additionally, the sunset funnel plot also illustrates the overall lack of power associated with many of the included studies, where around half have 50% power or less. All studies were given a quality score, ranging from 3 (Bridges, 1991; Calhoun et al., 1976) to 17 (Franklin & Garza, 2018). The mean quality score was 7.78, which is considerably below the maximum score of 22, indicating an overall low level of quality among the included studies. Analysis was carried out using the quality score as a moderator. The analysis indicated that study quality did not have an impact on the results in the current context, β = 0.02, *p* = .95, 95% CI [−0.07, 0.08].

### Descriptive Findings of the Studies

A summary of the descriptive features of the studies, including quality scores, can be found in Supplementary Table S1 (https://osf.io/7udxg/). An overall summary of the critical findings can be found in Table 1.

**Table 1.** Critical Findings.

Critical Findings
Victims of acquaintance rape are blamed more than victims of stranger rape, equating to a medium effect size. The closeness of this relationship does not appear to moderate this effect. In a minority of studies, victims of stranger rape face increased victim blame. It is likely that perceived victim precipitation (e.g., “risky” behavior) contributes to this. Rape myth acceptance (RMA) continues to be a relevant variable in research into victim blame but may explain overall victim blame better than differences between stranger and acquaintance rape. RMA should be treated as an attitudinal variable, rather than as a situational feature of the vignette itself. Ambivalent sexism appears to be the more theoretically relevant variable in the context of victim blame and victim–perpetrator relationship. Benevolent sexism in particular warrants further research. There is not enough transparency in research practices in this area, which limits the degree to which findings can inform policy and practice. Specifically, there is not enough information on how vignettes are developed and validated. This may partially explain past inconsistencies in this research area.

#### Victim–perpetrator relationship

The vast majority of included studies reported that a woman who knew her perpetrator was blamed more than a woman who did not. There are a number of possible explanations for why a small number of studies reported more victim blame in stranger rape scenarios. As proposed by Bolt and Caswell (1981), it is possible that participants view certain stranger rape situations as caused by the victim’s behavior, particularly if these involve the victim making “risky” choices, such as walking home alone at night in an area where previous rapes have occurred (e.g., Calhoun et al., 1976) or hitchhiking with a stranger, as opposed to hitchhiking with someone familiar (Idisis et al., 2008). A similar argument could be made regarding the stranger rape scenario in the study by Strömwall et al. (2013), where the stranger was in fact the friend of a friend who visited the party hosted by the woman in her home; it is possible that participants blamed the woman for allowing relative strangers into her home, rather than for the rape per se.

Within the acquaintance rape condition, there was mixed evidence concerning the different levels of familiarity between the victim and the perpetrator. Although some studies (e.g., Bieneck & Krahé, 2011; Bridges, 1991) reported a linear association between the victim–perpetrator relationship and victim blame (i.e., increased familiarity increasing assigned victim blame), a number of studies did not demonstrate a clear distinction between acquaintance and partner rape. While victims of acquaintance rape were consistently assigned more blame than victims of stranger rape, this blame did not always increase when the perpetrator was a partner (e.g., Newcombe et al., 2008; Simonson & Subich, 1999), and at times victims of partner rape were blamed less than victims of stranger rape (e.g., Bendixen et al., 2014; Hammock & Richardson, 1997; Hine & Murphy, 2019). A possible reason for why partner rape victims were at times viewed more favorably is that participants may consider it reasonable for a woman to feel safe with a partner, but not with a casual acquaintance. In fact, they may be of the view that it is the victim’s responsibility to not be trusting of a casual acquaintance.

Where the victim of partner rape was blamed for the assault (e.g., Bridges, 1991), it is possible that pre-assault behavior such as kissing is perceived as the woman “leading the man on” (aligning with central tenants of both AS and RMA regarding heterosexual relationships), possibly increasing attribution of responsibility to the victim. The above, therefore, calls for clarity regarding additional factors in rape vignettes that may have an impact on the way in which responsibility is assigned. Sexual behavior of the victim leading up to the assault appears to be an important factor in influencing perceived blame, so future research should aim to clarify whether this is present or not in the vignette. Ideally, if comparing stranger and acquaintance rape, victim sexual behavior should be kept to a minimum, as by definition, a stranger with whom the victim flirts or engages in sexual activity conceptually becomes an acquaintance.

#### Participant sex

Where sex differences were reported, a majority of the studies indicated that men had higher levels of victim blame than did women, both in the stranger and acquaintance rape conditions (e.g., Areh et al., 2009; Bell et al., 1994; Bendixen et al., 2014). A minority of studies found no such effect (e.g., Pedersen & Strömwall, 2013), but as noted by Krahé et al. (2008), it is possible that men’s higher levels of RMA account for an indirect effect on victim blame. Men’s higher levels of RMA were noted by a number of the studies included in the current data set (e.g., McKimmie et al., 2014; Murphy, 1992; Newcombe et al., 2008). This would be applicable across contexts, as a meta-analysis of 37 studies by Suarez and Gadalla (2010) found that men consistently endorse rape myths to a greater degree than do women. This contradicts evidence by Grubb and Turner (2012), who report limited sex differences in victim blame in rape cases. It does, however, support more recent findings by Hockett et al. (2016) who established that men assigned more blame to the victim than women did. Not all studies (Rodriguez et al., 2015) analyzed and/or reported sex differences in victim blame, calling for future research to consistently conduct and report these analyses.

#### RMA

A total of 18 studies measured RMA and its relationship with victim blame. These studies used a variety of measures to asses participants’ agreement with rape myths: five used the Illinois Rape Myth Acceptance Scale (IRMAS; Payne et al., 1999), another five used the Acceptance of Modern Myths about Sexual Aggression (AMMSA) scale (Gerger et al., 2007), five used the Rape Myth Acceptance Scale (RMAS; Burt, 1980), and three used the R Scale (Costin, 1985). All of these studies reported that RMA correlated with victim blame, something that is well-supported in previous synthesis of literature (Grubb & Turner, 2012; Suarez & Gadalla, 2010). As for the interaction between RMA and type of rape, none of the studies reported that RMA had an impact on the magnitude of the difference in blame attributions between conditions, suggesting that RMA does not influence the effect of the victim–perpetrator relationship. Specifically, those high in RMA blame the victim consistently across scenarios, and those low in RMA blame the victim to a lesser degree across scenarios. This suggests that the effect of the victim–perpetrator relationship is robust enough to not be impacted by RMA, and that the bias toward blaming the woman who knows her perpetrator is pervasive across degrees of RMA.

A difficulty when examining the influence of RMA on victim blame in different relationship contexts is the wide array of scales used, with a difference of nearly 30 years between the oldest and the most recent one. As has been noted by Bohner et al. (2009), some of the older scales (e.g., RMAS and Costin R Scale) have been criticized for lengthy wording and an overuse of archaic colloquialism, which may seem out of place in different contexts and times. An additional issue as noted with some of the older scales is the floor effect (i.e., highly skewed results) produced among younger samples, where means cluster at the low end (Gerger et al., 2007). This produces methodological issues when attempting to measure participants’ actual attitudes and not what they perceive to be socially acceptable (Hinck & Thomas, 1999; McMahon & Farmer, 2011). More recent scales such as the AMMSA (Gerger et al., 2007) and the updated IRMAS (known as the U-IRMAS; McMahon & Farmer, 2011) have attempted to account for the above issues by using subtler, less direct language. This review, therefore, recommends that future research adopts subtler, more recent scales to measure RMA.

#### AS

A minority of studies (*N* = 5) examined AS and its relation to victim blame, as measured by the ASI (Glick & Fiske, 1996). In line with the theoretical underpinnings of this construct, it appeared that BS and HS had different impacts on victim blame depending on the victim–perpetrator relationship. A central tenant of AS is that good women are regarded as deserving of protection, whereas bad women are to blame for bad things happening to them. This is consistent with the Just World Belief theory proposed by Lerner (1980). If a victim of acquaintance rape is viewed as being complicit in the assault (by, for instance, going on a date with the assailant), BS would posit that she is a bad woman, who deserved the negative consequences. This theory was confirmed by a majority of the studies; Pedersen and Strömwall (2013) and Yamawaki (2007) found that BS was a significant predictor of victim blame in the date rape scenario, but not the stranger rape scenario. Specifically, the higher someone scored on BS, the more they blamed the victim of acquaintance rape. Similarly, two studies from Abrams et al. (2003; Studies 1 and 2) found that BS predicted victim blame in their acquaintance condition, but not in the stranger condition, with the same pattern of direction. Conversely, Persson et al. (2018) found that HS and BS predicted levels of victim blame in the stranger condition, but not in the acquaintance condition. Overall, this suggests that AS is a key variable of interest when examining blame attributions in stranger and acquaintance rape cases, as it seems to tap into level of acquaintance as a factor for perceived victim precipitation. It would, therefore, seem highly relevant for future research into victim–perpetrator relationship to consider how victim blame interacts with AS in different contexts.

#### Relationship between rape myths and AS

Only three studies measured AS and RMA. Abrams et al. (2003; Studies 1 and 2) reported that RMA positively correlated with both HS and BS, with the former correlation being slightly stronger. These findings are similar to Persson et al. (2018) who reported identical patterns of positive correlations among the three variables. This echoes previous findings by researchers such as Chapleau et al. (2008) who found that both HS and BS correlated positively with RMA among a large sample of college students. That the correlations between RMA and HS are slightly stronger may be because both constructs tap into primarily aggressively sexist attitudes. While BS also supports the notion of traditional gender roles in sexual relationships (e.g., women serving as gatekeepers for sex) similar to RMA, it also stresses women’s moral superiority and pureness as a reason for this, which RMA does not. The above, therefore, suggests that although BS may ostensibly be perceived as less harmful than HS, it actually contributes to victim blame and RMA. This supports contentions by Glick and Fiske (1996), in that all forms of prejudice against women are harmful for gender equality and that sexism in all forms need to be challenged.

#### Study quality

Papers were graded using a quality score tool, which resulted in most studies failing to be considered to have achieved even half of the criteria. Studies were generally poor at accounting for missing data and confounds as well as reporting the analysis details clearly and comprehensively. Sample groups were also rarely matched (or compared on key demographics) or reported in the context of a power analysis. The low levels of power across some of the study samples were also highlighted in the sunset funnel plot (Kossmeier et al., 2020), although there were also good examples of well-powered research. The above, therefore, somewhat limits the conclusions that can be drawn from the current review. Examples of good research practice included the use of reliable dependent measures and moderators and recruitment of nonstudent samples (e.g., prospective lawyers [Krahé et al., 2008]; police officers [Hine & Murphy, 2018]; and members of the community [Sommer et al., 2016]). This review concludes that there is a need for more comprehensive methodology in the current area of research and that studies need to account for key aspects of study design and, most importantly, consider the way analysis is carried out and reported.

Relatedly, although vignettes had generally been implemented in more than one study, there was little information on any validation process for these, which is problematic. Specifically, there was no information available on whether participants found these vignettes believable and whether they were realistic enough to elicit similar attributions as a real-life case would do. Therefore, there is a need for increased transparency in how rape vignettes are developed and for the implementation of validated vignettes that are considered believable by participants. This would increase the utility of this research for practitioners and policy makers.

## Discussion

The current review and meta-analysis provide a valuable contribution to the current literature on blame attributions in rape cases in several ways. First, although previous reviews (e.g., Grubb & Turner, 2012) have established a difference in victim blame attributions between stranger and acquaintance rape, this article quantifies this by adding a meta-analysis to support its contentions, and including a considerable number of more recent studies. It also addresses some of the inconsistencies highlighted in previous research (Grubb & Harrower, 2009), particularly in systematically examining attitudes to gender and quantifying methodological quality across studies. By doing so, it is possible to make concrete recommendations for future research in this area, which will improve the capacity for sexual assault research to have practical and policy implications. Second, while previous studies have examined RMA as a moderating variable (Gravelin et al., 2018; Hockett et al., 2016), the current study treats RMA as an attitudinal variable, rather than as a feature of the rape case itself. It also expands the consideration of RMA beyond acquaintance rape only. Third, in addition to examining the moderating influence of RMA, this article also considers the impact of AS, which appears to be the most relevant variable when considering blame attributions in different victim–perpetrator relationships. The systematic review provides evidence for the importance of BS in particular. Finally, this meta-analysis accounts for the interdependency of ESs from the same studies with unknown correlations by applying a three-level meta-analytical model, made possible by the Metafor package (Viechtbauer, 2010) in R. This means that while the focus of this study is novel in itself, it also furthers the analytical approach in conducting meta-analyses in this area.

The results of the analyses indicate that there is a medium effect of acquaintance rape victims being blamed to a greater degree than victims of stranger rape. This is in line with previous reviews (e.g., Grubb & Turner, 2012) and indicates that a woman who knows her perpetrator even in the slightest is blamed significantly more for the assault than someone who does not know her perpetrator. This is extremely worrying given that most women who are raped know the perpetrator, which is likely to have an impact on both how the rape is perceived by women subjected to sexual violence and observers in public or professional roles (Du Mont et al., 2003). It is possible that the increased levels of victim blame in acquaintance rape scenarios stem partly from a misconception among the public about the typical features of a rape, where stranger rape is seen as the norm (and, therefore, more “legitimate” or a “real rape”), and anyone who knows her perpetrator deviates from this (Smith, 2018). That the public holds incorrect perceptions about typical victim and perpetrator behavior during a rape has been established in research on stranger rape by Sleath and Woodhams (2014), although it is unclear how this translates in acquaintance rape scenarios. Future research may, therefore, wish to examine how knowledgeable participants, including professionals, are about acquaintance rape more generally. Finally, the current study did not find a moderating effect of level of acquaintance, in that a closer relationship to the perpetrator did not automatically result in more attributed blame. As previously mentioned, it is possible that this has less to do with level of acquaintance and more to do with perceived victim precipitation before the assault. The current review, therefore, calls for increased clarity about victim behavior in vignette details and for the consistent reporting of manipulation checks (i.e., the degree to which key study manipulations are implemented correctly).

It further appears that RMA does not moderate the magnitude of the effect of the victim-perpetrator relationship, as was supported by both the systematic review and the meta-analysis. This is likely because those high in RMA will attribute blame to victims of rape regardless of the relationship between victim and perpetrator (Suarez & Gadalla, 2015), without paying too much attention to particular case details. It is, therefore, likely that the key variable of interest in this context is AS, as the systematic review indicated a more complex relationship between this and victim blame. Specifically, it appears that those high in BS will attribute more blame to the victim of acquaintance rape than stranger rape.

It is important to note that the measurement of victim blame varied substantially in the literature. Some researchers assessed “blame,” others assessed perceived “responsibility,” and one “guilt.” Although often treated as synonymous, previous research (Shaver & Drown, 1986) indicates that when participants are asked to attribute blame, responsibility, and causality to a variety of different individuals and situations, responsibility was attributed more than blame and causality. This is perhaps because blame may be a harsher assessment than responsibility, resulting in participants being more comfortable attributing responsibility than blame (Gravelin et al., 2018). However, the literature is inconsistent with respect to this (for a review, see Gravelin et al., 2018), and the current meta-analysis did not find that type of outcome measurement moderated the overall effects, which is similar to findings by Hockett et al. (2016). Therefore, in the current context, it appears that victim blame/responsibility/guilt/culpability can be used relatively interchangeably.

This review makes a number of recommendations relevant to anyone working to develop practice or policy to improve provisions and justice for rape victims. Relatedly, it also makes a number of recommendations to future researchers in this area. These recommendations can be found in Table 2.

**Table 2.** Review Implications.

Review Implications	
Practice & policy	Acquaintance rape victims are generally blamed more than stranger rape victims, but this is not always true and may depend on perceived risky behavior of the victim. Relatedly, perceived victim precipitation (e.g., flirting) appears to increase victim blame, which taps into commonly believed rape myths. This should be considered when providing support to victims of rape and also when ensuring successful prosecution strategies. High rape myth acceptance (RMA) appears to increase victim blame generally (rather than moderate any other relationships), which can reduce chances of successful prosecutions and justice for rape victims. These attitudes need to be challenged on a societal level, which is of particular importance to those working within education. Ambivalent sexism may seem harmless, but benevolent sexism in particular appears to punish victims of acquaintance rape. Policy makers must challenge general differentiations between women and men. Policy recommendations need to be based on robust research. Research in this area needs improved methodology to inform real-life situations.
Future research	RMA should be treated as an attitudinal variable rather than as a situational feature of the rape scenario. Ambivalent sexism may be able to explain inconsistencies in past research and should be further investigated in the context of victim-perpetrator relationship and victim blame. Sexual assault vignettes need to be consistent in terms of keeping confounding variables constant. Research methodologies need to be improved. This includes transparency in vignette development and research practices. Adhering to Open Science principles (e.g., making datasets openly available, validating vignettes, etc.) can achieve this.

### Limitations and Future Directions

There were a number of limitations associated with the current review that should be considered when interpreting the results. A major limitation of the current study is the restricted diversity of the included studies; the vast majority of studies were conducted in Europe, United States, and Australia, with only three conducted in Asia. Consequently, none of the reviewed studies were conducted in Africa or Latin America, significantly limiting our knowledge about how women who have experienced rape are perceived in non-Western cultures. This is particularly relevant in the current context, as a considerable number of international countries (e.g., Iran) do not acknowledge marital rape as a crime, presumably having an impact on the way in which victims of acquaintance rape are perceived (Westmarland & Gangoli, 2011). This, therefore, suggests that future research may wish to look beyond non-Western contexts when examining victim blame attitudes, particularly in how they interact with RMA and AS.

Further, the methodological quality of the included studies limits the conclusions that can be drawn from the synthesis of results. On average, the studies scored well below the midpoint for possible methodological quality, indicating that there is a considerable need for rigor concerning study design and execution within this area of research. While there were numerous examples of well-designed research (particularly in terms of sample recruitment), aspects such as statistical analysis and reporting need to be improved. This is of concern, as it makes it difficult to assess the validity of claims made about the results of the research, naturally having an impact on the overall results of the current study as well. This article calls for further methodological rigor, for instance by employing Open Science principles, in future research into blame attributions in rape cases.

Finally, this review acknowledges the limitations of the current research’s focus on negative rape-related outcomes, as it may enforce oppressive structures about gender and unfairly impose victimhood on women who have been raped. As noted by Hockett and Saucier (2015), focusing on the experience of survival and post-assault growth may encourage better outcomes for women who have been raped, both in terms of individual responses and public perceptions. It may, however, detract from the considerable negative consequences associated with rape, as noted within the “Victim-Survivor Paradox” (Thompson, 2000), indicating that future research into this topic may wish to consider more nuanced ways to examine individual responses to rape.

## Conclusions

This review and meta-analysis provide a comprehensive insight into variations in victim blame across victim–perpetrator relationships. The synthesis of results established a medium ES of victim–perpetrator relationship, where women assaulted by someone they knew were blamed more than those who did not know the perpetrator. RMA was associated with overall increased levels of victim blame but did not moderate the overall main effect. The review demonstrated that AS may be the more theoretically relevant variable when examining differences in blame attributions across scenarios with differing victim–perpetrator relationships. While the methodological quality of the included studies and the relative homogeneity of research contexts may impact conclusions that can be drawn, the current study does nonetheless contribute to the current literature on rape-related attitudes, most specifically on the importance of the victim–perpetrator relationship on blame attributions in rape cases and on the need to employ a rigorous and transparent methodology in this area overall.

## Supplemental Material

Supplemental Material, sj-pdf-1-tva-10.1177_1524838020977146 - Attributions of Blame in Stranger and Acquaintance Rape: A Multilevel Meta-Analysis and Systematic Review Click here for additional data file. Supplemental Material, sj-pdf-1-tva-10.1177_1524838020977146 for Attributions of Blame in Stranger and Acquaintance Rape: A Multilevel Meta-Analysis and Systematic Review by Sofia Persson and Katie Dhingra in Trauma, Violence, & Abuse
