# Supplementary material for: Attributions of Blame in Stranger and Acquaintance Rape: A Multilevel Meta-Analysis and Systematic Review
Source: Trauma Violence Abuse. 2020 Dec 7;23(3):795–809. doi: 10.1177/1524838020977146 (PMC9210121; doi:10.1177/1524838020977146)
Supplement: Supplemental Material, sj-pdf-1-tva-10.1177_1524838020977146 - Attributions of Blame in Stranger and Acquaintance Rape: A Multilevel Meta-Analysis and Systematic Review [file sj-pdf-1-tva-10.1177_1524838020977146.pdf]

Table S1. Descriptive Features of the Studies.

Study	Year	Country	Sample Type	Gender	Moderator	Level of Acquaintance	Quality Score
Abrams *et al.* (Study1)	2003	UK	Students	Mixed	RMA; AS	Acquaintance	15
Abrams *et al.* (Study 2)	2003	UK	Students	Male	RMA; AS	Acquaintance	12
Alexander	1980	US	Nurses	Mixed	-	Acquaintance; husband	10
Areh, Gorazd & Umek	2009	Slovenia	Legal	Mixed	-	Husband	8
Ayala, Kotary & Hetz	2018	US	Students	Female	RMA	Acquaintance	12
Barnett *et al.*	1992	US	Students	Mixed	-	Acquaintance	7
Bell, Kuriloff & Lottes	1994	US	Students	Mixed	-	Date	8
Bendixen *et al.*	2014	Norway	Community	Mixed	RMA	Acquaintance; partner	9
Bieneck & Krahé	2011	Germany	Student	Mixed	-	Acquaintance; ex	9
Bolt & Caswell	1981	Germany	Student	Female	-	Acquaintance	4
Bridges	1991	US	Student	Mixed	-	Date; partner	3
Bridges & McGrail	1989	US	Student	Mixed	-	Date; partner	0
Calhoun, Selby & Warring	1976	US	Student	Mixed	-	Acquaintance	3
Cowan	2000	US	Students	Mixed	-	Acquaintance, partner	7
Franklin & Garza	2018	US	Students	Mixed	RMA	Acquaintance	17
George & Martinez	2002	US	Students	Mixed	-	Acquaintance	10
Gerdes, Dammann & Heilig	1998	US	Students	Mixed	-	Acquaintance	3
Gölge *et al.*	2003	Turkey	Students	Mixed	-	Date	5
Hammock & Richardsen	1998	US	Students	Mixed	-	Date; partner	
Hine & Murphy	2009	UK	Police	Mixed	RMA	Acquaintance; partner, ex	8
Howells *et al.*	1984	UK	Community	Mixed	-	Acquaintance	3
Idisis, Ben-David & Ben-Nachum	2007	Israel	Mixed	Mixed	-	Acquaintance	2

Study	Year	Country	Sample Type	Gender	Moderator	Level of Acquaintance	Quality Score
Johnson & Russ	1989	US	Students	Mixed	-	Date	4
Krahé *et al*. (Study 1)	2008	Germany	Students	Mixed	RMA	Acquaintance; ex	8
Krahé *et al*. (Study 2)	2008	Germany	Legal	Mixed	RMA	Acquaintance; ex	7
Krahé, Temkin & Bieneck (Study 1)	2007	Germany	Students	Mixed	-	Acquaintance; ex	7
Krahé, Temkin & Bieneck (Study 2)	2007	Germany	Students	Mixed	-	Acquaintance; ex	7
L'Armand & Pepitone	1982	US	Students	Mixed	-	Acquaintance; ex	1
McKimmie, Masser & Bongiorno	2014	Australia	Community	Mixed	RMA	Acquaintance	10
Murphy	1992	US	Students	Mixed	RMA	Acquaintance; partner, ex	6
Newcombe *et al.*	2008	Australia	Students	Mixed	RMA	Acquaintance; date; partner	7
Pedersen & Strömwall	2013	Sweden	Community	Mixed	AS	Acquaintance	10
Persson, Grogan & Dhingra	2018	Sweden	Mixed	Mixed	RMA; AS	Acquaintance	8
Quackenbush	1989	US	Students	Male	RMA	Acquaintance	6
Rodriguez, Berry & Gresley	2015	US	Students	Mixed	RMA	Acquaintance	7
Sasson & Paul	2015	US	Community	Mixed	RMA	Acquaintance	4
Scronce & Corocan	1994	US	Students	Mixed	-	Acquaintance	4
Simpson & Subich	1991	US	Students	Mixed	-	Acquaintance: date; partner	11
Sommer, Reynolds & Kehn	2016	US	Community	Mixed	RMA	Acquaintance: partner	8
Stacy, Prisbell & Tollesfrud	1992	US	Students	Mixed	-	Date	5
Strömwall, Alfredsson & Landström	2013	Sweden	Community	Mixed	-	Acquaintance: partner	8
Szymanski *et al.*	1993	US	Students	Mixed	RMA	Acquaintance	5
Tetreault & Barnett	1987	US	Students	Mixed	-	Acquaintance	4
Willis	1992	US	Students	N/A	-	Date	2
Yamawaki	2007	US	Students	Mixed	AS	Partner	6
Yamawaki	2009	Japan	Students	Mixed	RMA	Date	9
Yuvarajan & Stanford	2016	US	Clergy	Mixed	-	Acquaintance; date; partner	3

Non-Significant Moderator Analyses.

Type of relationship: F (3,44) = 0.72, p = .54.

Percentage of women in sample: β = 0.01, p =.84, 95% CI = [-0.02, 0.01].

Age of sample: β = 0.00, p = .99, 95% CI = [-0.03, 0.03].

Level of RMA: β = 0.07, p =.97, 95% CI = [-3.6, 3.7].

Type of outcome variable (victim blame and victim responsibility): F (1,48) = 0.41, p = .52

Year of study publication: β = -0.004, p = .68, 95% CI = [-0.025, 0.02].
